# Targeting surface cell antigen 2 increases sensitivity of *Rickettsia typhi* detection

**DOI:** 10.1371/journal.pntd.0014004

**Published:** 2026-02-19

**Authors:** Weerawat Phuklia, Kaisone Padith, Koukeo Phommasone, Vilada Chansamouth, Mayfong Mayxay, Susath Vongphachanh, Paul N. Newton, Matthew T. Robinson, Elizabeth A. Ashley

**Affiliations:** 1 Lao-Oxford-Mahosot Hospital-Wellcome Trust Research Unit (LOMWRU), Microbiology Laboratory, Mahosot Hospital, Vientiane, Lao People’s Democratic Republic; 2 Nuffield Department of Medicine, Centre for Tropical Medicine & Global Health, University of Oxford, Oxford, United Kingdom; 3 Institute of Research and Education Development (IRED), University of Health Sciences, Ministry of Health, Vientiane, Lao People’s Democratic Republic; 4 Mahosot Hospital, Vientiane, Lao People’s Democratic Republic; 5 Mahidol-Oxford Tropical Medicine Research Unit, Faculty of Tropical Medicine, Mahidol University, Bangkok, Thailand; State Key Laboratory of Pathogen and Biosecurity, CHINA

## Abstract

**Background:**

Murine typhus is a flea-borne disease caused by *Rickettsia typhi* that typically presents as an acute febrile illness. The diagnosis is often missed, leading to delays in appropriate treatment. A qPCR targeting a single gene (*ompB)* for *R. typhi* is widely used for diagnosis; however, it has low sensitivity for detecting bacterial DNA in patients’ blood.

We aimed to increase sensitivity of detection of *R. typhi* using qPCR by targeting a gene containing repetitive sequences, *sca2* (surface cell antigen 2)*.*

**Methodology:**

We compared diagnostic accuracy with the standard assay targeting single sequence *ompB* (outer membrane protein B). Specificity, sensitivity, and bacterial load measurement of both assays were compared using stored EDTA-anticoagulated buffy coat samples from 88 patients with febrile illness at Mahosot Hospital or provincial hospitals in Laos. Among these, 55 cases were confirmed as positive or negative by first culturing *Rickettsia* spp. from patients’ EDTA blood, followed by assessment with IFA and *ompB* PCR, and buffy coat from 6 additional cases was confirmed by *ompB* PCR. Ten further positive cases were confirmed by IFA using paired sera, and 17 cases classified as negative for scrub typhus and murine typhus based on Rapid Diagnostic Test (RDT) results were included in the evaluation.

**Results:**

The *sca2* qPCR assay showed 59.09% sensitivity (95% CI, 38.73-76.74%) and 100% specificity (93.98-100%) for detection of *R. typhi*. In comparison, *ompB* assay demonstrated 36.36% sensitivity (95% CI, 19.73-57.05%) and 100% specificity (95% CI 93.98-100%). DNA copy number determined using the *sca2* gene was approximately 2.933 log unit higher than that determined using *ompB* gene (median, 16,500 copies/μL; IQR, 13,045–40,000 versus median, 19.25 copies/μL; IQR, 11.11-56.62, P < 0.0001).

**Conclusion:**

This study suggests qPCR targeting *sca2* increases frequency of detection of *R. typhi* in patients with low bacterial DNA concentrations.

## Author summary

*Rickettsia typhi* is a bacterium that causes murine typhus. This disease is often overlooked because its symptoms are very similar to those of other infections. It can also be hard to detect because patients usually have a low blood bacterial concentration, making it difficult for current assays to detect. We focused on a part of the bacteria called *sca2* (surface cell antigen 2), which has repeating sections, thinking that looking for these repeating sections could make molecular detection methods more sensitive, allowing them to detect even very low levels of the bacteria. We designed a new test that targets these repeating sections of the *sca2* gene and tested it using different strains of *R. typhi* and related bacteria. Compared to the conventional test that looks for the *ompB* (outer membrane protein B) gene, the new *sca2* test was able to detect lower amounts of the bacteria in patient samples. This means it could be a more effective tool for diagnosing the disease, especially when the amount of bacteria in a patient’s blood is very low.

## Introduction

*Rickettsia typhi* is an obligate intracellular bacterium that causes the neglected disease murine typhus. Both the rat flea (*Xenopsylla cheopis*) and the cat flea (*Ctenocephalides felis*), play a role in transmitting the disease [1]. The disease has a worldwide distribution, including in the Lao PDR (Laos) [2]. Although the disease is frequently mild and responds to key antibiotics, the mortality rate in untreated patients is approximately 0.4% [3]. Misdiagnosis often leads to inappropriate treatment. In the absence of a single gold diagnostic standard, a combination of serology and PCR performed on acute samples is widely accepted for the diagnosis of murine typhus. However, some serological methods cannot provide results quickly enough for early clinical decision-making [4–6]. In contrast, quantitative PCR (qPCR) targeting *R. typhi*-specific genes is a valuable tool for early diagnosis, as it can detect bacterial DNA in acute patient samples [7]. The outer membrane protein B (*ompB*) gene is commonly used as a target for *R. typhi* detection [8]. However, it targets a single-copy region of the gene and its sensitivity may be limited, especially in patients with a low bacterial load [9]. In the case of *Orientia tsutsugamushi*, another obligate intracellular bacterium, targeting repetitive gene sequences has been shown to improve diagnostic sensitivity [10]. We therefore proposed applying a similar strategy for murine typhus.

Surface cell antigen (sca) proteins belong to the autotransporter protein family in *Rickettsia*. The *R. typhi* genome encodes five Sca proteins; sca1, sca2, sca3, sca4 and sca5. These proteins are essential for bacterial survival. Although *R. typhi* contains relatively few repetitive sequences in its genome [11], the *sca2* gene has six tandem repeats within a 150-base-pair region. This protein may facilitate bacterial adhesion to host cells [12]. Given its essential role in *R. typhi*’s intracellular replication, the *sca2* gene represents a promising new target for diagnostic assays. In this study we aimed to develop a qPCR assay for *R. typhi* detection by targeting a gene region containing repetitive sequences (*sca2*) and to compare its performance for detection of *R. typhi* with the existing qPCR assay that targets *ompB*.

## Methods

### Ethics statement

These samples were part of previous studies that were approved by the National University of Laos, National Ethics Committee for Health Research (NECHR) and National Institute of Public Health (NIOPH), Vientiane and Oxford Tropical Research Ethics Committee (OxTREC), Faculty of Medical Sciences. For all studies, written informed consent was obtained from all adult participants and from parents or legal guardians of child participants prior to sample collection.

### Assay development

#### Searching for repetitive sequences.

The *sca2* gene, encoding the 190-kDa surface cell antigen (*sca2*) of *Rickettsia typhi* strain Wilmington, was identified from the complete genome sequence (GenBank accession number AE017197.1) at genomic positions 68,152–72,603 and corresponds to locus tag RT0052. To investigate repetitive sequence within the *R. typhi sca2* gene, the full length of nucleotide sequence (4,452 bp) was submitted to the Tandem Repeat Finder (https://tandem.bu.edu/trf/trf.basic.submit.html). The sequence was analyzed as described [13] and four regions containing repetitive sequences are illustrated in S1 Fig. The region with the highest number of repetitive sequences was selected as the target for PCR detection. Primers were designed to amplify this region using Primer 3 (https://bioinfo.ut.ee/primer3-0.4.0/), as shown in S1 Fig. PCR amplification using these primers generates products whose size may vary depending on the number of repeat copies in the target region. The primer sequences were sca2F 5’- TGGAATGGACAGTAAAACGACAG-3’ and sca2R 5’- CGTTCTGCTGCCTCTTCTGA-3’.

#### Determination of assay specificity.

To test the specificity of the selected primers, stored DNA from several strains of cultured *R. typhi* (Wilmington, AZ331 and GER), as well as DNA from other bacteria including *Rickettsia prowazekii*, *Rickettsia felis*, *Rickettsia conorii*, *O. tsutsugamushi*, *Burkholderia thailandensis*, *Burkholderia cepacia*, *Anaplasma phagocytophilum*, *Leptospira* spp., and *Neorickettsia sennetsu* were subjected to PCR and the bands were observed using 2% agarose gel electrophoresis.

#### *R. typhi* quantification by quantitative real time PCR (qPCR).

DNA from *R. typhi* culture or patients’ buffy coat was extracted using GeneJet Genomic DNA purification kit (ThermoFisher Scientific, UK), following the manufacturer’s instructions. The DNA copy number of *R. typhi* was measured using qPCR targeting either *sca2* or *ompB*. The primers for *sca2* were described above, and the Taqman probe for the *sca2* gene was sca2P: 5’-FAM- ACAGATAACATAGCAGCAGAATCT-BHQ1–3’. The primers and Taqman probe for *ompB* were Rt557F: 5’- TGGTATTACTGCTCAACAAGCT-3’, Rt678R: 5’-CAGTAAAGTCTATTGATCCTACACC-3’ and Rt640 BP: 5’-FAM- CGCGATCGTTAATAGCACCAGCATTATCGCG -BHQ1–3’. The qPCR mixture was composed of 1X qPCRBIO Probe Mix (qPCR Probe MIX LO-ROX, PCR Biosystems, UK), 0.4 μM forward and reverse primers, 0.2 μM probe, sterile distilled water and 1μL of extracted DNA. The *sca2* qPCR assay was performed in the presence of 0.4 μg/mL bovine serum albumin (BSA) per reaction to improve amplification efficiency. BSA helps stabilize the DNA polymerase and mitigate the inhibitory effects of secondary structures often present in repetitive sequences, enhancing reproducibility and sensitivity of the assay [14,15]. Real-time PCR was performed on a CFX96 real-time PCR detection system (Bio-Rad Laboratories) using the following conditions: initial denaturation at 95°C for 2 min, followed by 45 cycles of denaturation at 95°C for 15 sec and combined annealing and extension at 60°C for 30 sec with the acquisition of fluorescence. Rickettsial DNA copies per ml of blood were calculated using 10-fold serial dilution of known concentrations of *R. typhi sca2* and *ompB* fragments cloned into the pGEM-T Easy vector (ranging from 10^6^ to 10^0^ copies/μL).

#### Limit of detection (LOD) for qPCR assays using *R. typhi* spiked blood.

Limit of detection (LOD) is defined as the minimum number of DNA copies in a sample that can be reliably detected with the assay. We used the plaque assay, the gold standard for *Rickettsia* quantification [16], to determine rickettsial load in terms of plaque forming units (pfu). *R. typhi* strain Wilmington was used to spike whole blood collected from healthy donors (n = 2). Purified *R. typhi* was serially diluted ten-fold and spiked into blood samples. Specifically, 30μL of media containing *R. typhi* at concentrations ranging from 10^1^-10^6^ pfu/mL was added to 270 μL of blood. The resulting 300 μL mixture containing *R. typhi* ranging from 3 x 10^-1^ to 3 x 10^4^ pfu/total blood was incubated at room temperature for 30 minutes, after which DNA was extracted from 300 μL of blood using GeneJet Genomic DNA Purification Kit as described by manufacturer’s instruction (ThermoFisher Scientific, UK). *R. typhi* DNA in each dilution was quantified using both the *ompB* and *sca2* qPCR assays.

### Diagnostic accuracy testing

#### Clinical samples.

Three groups of stored buffy coat samples were used to validate and compare the specificity and sensitivity of the *sca2* and *ompB* assays. Relevant studies included “A multicentre, open-label randomized trial comparing 3-day doxycycline, 7-day doxycycline, and 3-day azithromycin for scrub and murine typhus” (OxTREC no. 003–03 and 1413/FMS), “The pathophysiology of typhus in Laos” (OxTREC no. 015–04 and 010/NECHR) and “A cross-sectional prospective study to identify the pathogenicity of acute non-malaria febrile illness” (OxTREC no. 015–10 and 134/NECHR). Samples in this group were confirmed by indirect immunofluorescence assay (IFA) and *ompB* PCR from cell culture or by *ompB* PCR from buffy coat. The second group of samples came from patients confirmed to have murine typhus (n = 10) from a fever surveillance study in three provinces in Laos (OxTREC no. 027–14 and 026 NIOPH/NECHR). Diagnosis in this group was confirmed by IFA using paired sera (acute and convalescent). The third group consisted of buffy coat samples from patients (n = 17) who tested negative for murine typhus RDT (ImmunoDOT *Rickettsia typhi* test (GenBio)) and scrub typhus by RDTs (Scrub Typhus Detect IgM Rapid System (Dipstick) (InBios)) in a prospective study of the causes of fever amongst hospitalized patients in Lao PDR (Tables 1 and S1).

**Table 1:** Repetitive sequence regions of *sca2* gene investigation.

Position	Size	Copies number	Consensus size	% matches	Percent Indel	Score	A	C	G	T	Entropy (0–2)
106-132	12	2.3	12	100	0	54	25	66	0	7	1.17
647-882	129	1.8	129	83	3	314	47	9	14	28	1.76
646-917	129	2.1	128	80	5	298	46	9	15	28	1.77
2360-3262	150	6.0	150	99	0	1779	47	13	20	18	1.82

Four repeat regions of *sca2* gene were observed using Tandem Repeat Finder (https://tandem.bu.edu/trf/trf.html).

#### DNA extraction.

DNA was freshly extracted from the frozen buffy coat samples using the GeneJet Genomic DNA Purification Kit (ThermoFisher Scientific, UK), following the manufacturer’s instructions. Briefly, 200 μl of the buffy coat was lysed and precipitated with ethanol, and the mixture was passed through a spin column for DNA binding. The final elution volume was 100 μl per sample. DNA was stored at −80°C for long-term preservation and at 4°C between qPCR runs.

### Data analysis

The sensitivity and specificity of the qPCR assay targeting *ompB* and *sca2* were calculated. Statistical significance was tested by Fisher’s exact test. The 95% confidence interval of sensitivity and specificity were calculated using Wilson-Brown method. As the qPCR copy number data were not normally distributed and included outliers, median and interquartile range (IQR) of Rickettsial loads determined by both assays was compared using Wilcoxon test. Graphical representations were calculated and generated using GraphPad Prism (version 10).

## Results

### Repetitive sequences on *R. typhi sca2* sequence

Repetitive sequences were identified at four distinct regions along the full-length *R. typhi sca2* gene. These regions contain approximately 2.3, 1.8, 2.1 and 6 copies repetitive elements, respectively, with varying sequence lengths, as summarized in Table 1. Among these, the region located at position 2360–3262 had the highest number of repeats (6 copies) and the largest sequence size. Due to its high repeat number and sequence length this region was selected as the target for qPCR assay development.

### Specificity testing

To evaluate the specificity of the designed primers for *R. typhi*, we first conducted *in silico* analysis comparing *R. typhi* strains to other *Rickettsia* species. The analysis demonstrated that the primers were specific to *R. typhi* genomes and did not amplify sequences from other species, including *R. prowazekii*.

We then validated these findings experimentally by performing conventional PCR using DNA extracted from *R. typhi* strains Wilmington, AZ331, and GER, as well as from other bacterial species. The result confirmed that *sca2* assay specifically amplified DNA from *R. typhi* strains, with no amplification observed in non-*R. typhi* samples (S2 Fig).

### Optimization of qPCR targeting *sca2* assay and *ompB*

**Fig 1:**
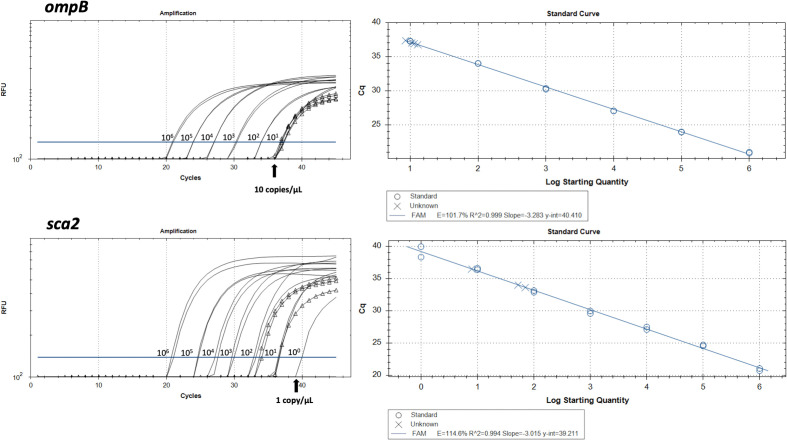
Standard curve for *Rickettsia typhi* qPCR assay. Assay used plasmid (pGEM-Teasy) carrying *ompB* or *sca2* fragment of *R. typhi* as target sequences, with concentrations ranging from 1 to 10^6^ copies/μL. The line with the blank triangle represents patient samples.

To determine the limit of detection (LOD) for both qPCR assays, serial 10-fold dilutions (10^6^ to 10^0^ copies/μL) of *R. typhi sca2* and *ompB* fragments cloned into pGEM-T Easy vector were used. The result showed that the LOD for the *ompB* assay was 10 copies/μL, while the sca2 assay demonstrated higher sensitivity with a LOD of 1 copy/μL (Fig 1).

### Limit of detection for qPCR assays targeting *ompB* or *sca2* using *R. typhi* spiked blood

**Fig 2:**
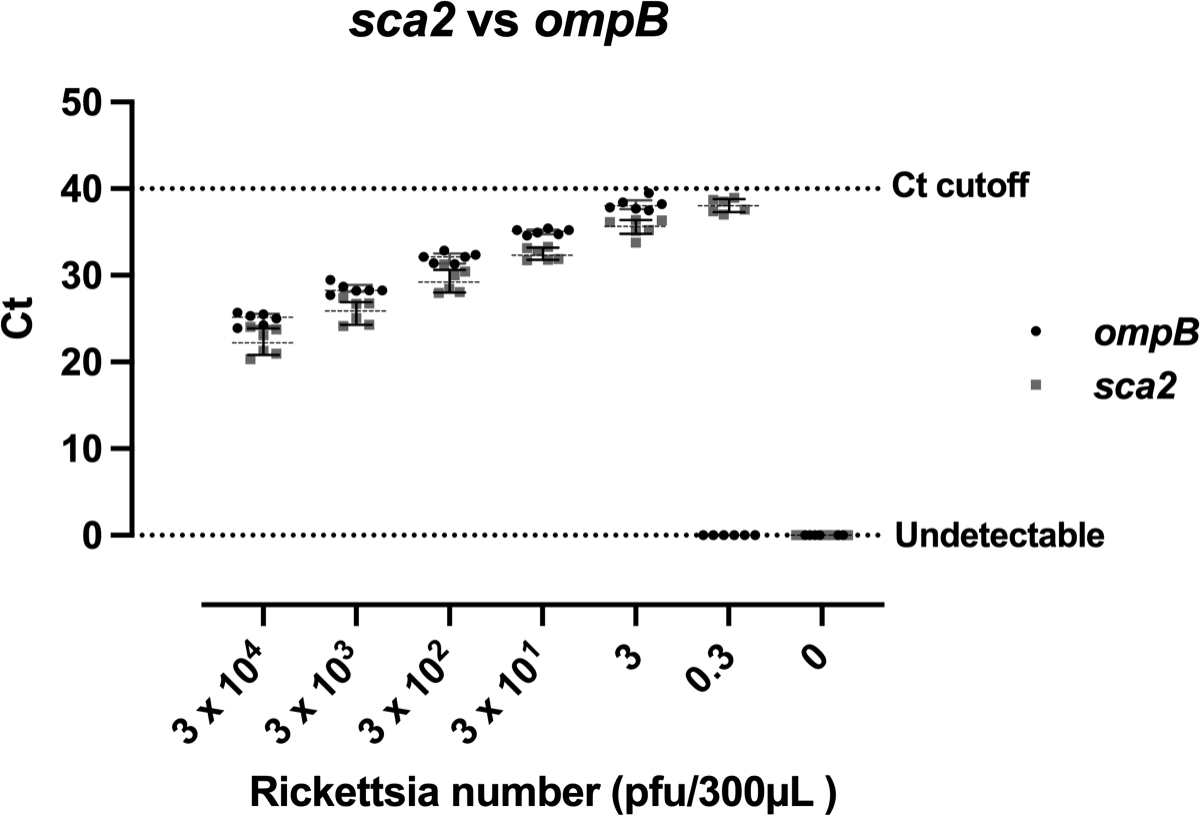
Limit of detection of *sca2* and *ompB* qPCR assay. *R. typhi* ranging from 0.3 to 3 x 10^4^ PFU of 30 μL of culture media containing *R. typhi* was spiked into 300 μL of whole blood from two healthy donors. Whole blood without *R. typhi* was included as the 0-PFU control. *R. typhi* was detected using different two assays targeting the *sca2* (grey) and *ompB* (black) genes. Each dot represents a Ct value obtained from qPCR for *sca2* or *ompB*. Dashed lines indicate the median with interquartile range (IQR) as error bars. Experiments for each gene were performed in triplicate per donor. The horizontal dashed line at Ct = 40 indicates the positivity cut-off (Ct ≤ 40).

We also compared the limit of detection between *ompB* and *sca2* assays by spiking whole blood with partially purified *R. typhi*. The result showed that qPCR using *ompB* was able to detect *R. typhi* 3 pfu/ 300 μL of whole blood whereas *sca2* was able to detect *R. typhi* 0.3 pfu/mL as demonstrated in Fig 2 and raw data for Cycle threshold (Ct) value are provided in S1 Data.

### Sensitivity and specificity testing of *sca2* and *ompB*

Clinical samples were subjected to both qPCR assays to compare their sensitivity and specificity. Raw qPCR Ct values and IFA results are provided in S1 Data. The results showed that the sensitivity of *sca2* assay was 59.09% (95% CI, 38.73-76.74%) and specificity was 100% (95% CI, 93.98-100) whereas the sensitivity of the *ompB* assay was 36.36% (19.73-57.05%) and specificity was 100% (95% CI, 93.98-100%) (Table 2)

**Table 2:** Sensitivity and specificity of *sca2* and *ompB* qPCR assays testing.

Buffy coat samples (n = 82)	*sca2* (%)	95% CI	*ompB* (%)	95% CI
**Sensitivity**	59.09%	38.73-76.74%	36.36%	19.73-57.05%
**Specificity**	100%	93.98-100%	100%	93.98-100%

Sensitivity and specificity of *sca2* and *ompB* qPCR assays on stored clinical samples from culture-positive and culture-negative cases, where culture status was confirmed by IFA and *ompB* PCR (n = 55), by IFA using paired sera (n = 10), or by rapid diagnostic test (n = 17)

### Rickettsial target gene copy number determination from positive *R. typhi* buffy coat by *ompB* PCR

**Fig 3:**
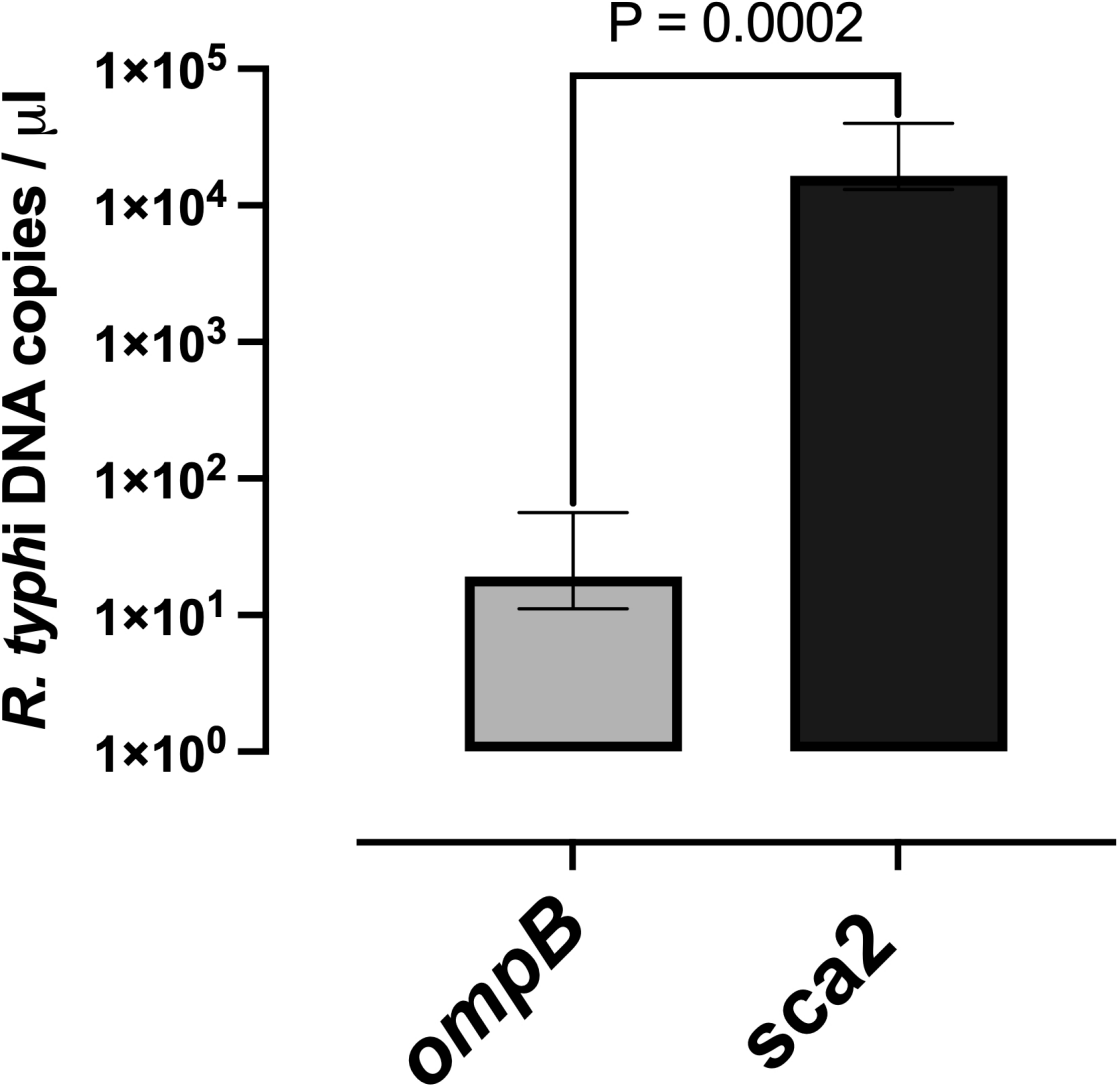
Comparison of Rickettsia target gene copy number using qPCR targeting *ompB* and *sca2.* Bacterial DNA from buffy coat samples (n = 13) were determined using *ompB* assay (grey) and *sca2* assay (black). Grey and black bar represent the median *Rickettsia* target gene copy numbers with interquartile range (IQR). Difference between assays were compared using Wilcoxon signed-rank test. A p-value <0.05 was considered statistically significant.

In order to compare rickettsial target gene copy number in buffy coat detected by *sca2* and *ompB* PCR, we calculated the copy number for both targets using *R. typhi sca2* or *ompB* fragments cloned into the pGEM-T Easy vector at concentrations ranging from 10^0^ to 10^6^ copies/μL. The median rickettsia target gene copy number detected using *sca2* gene was 16,500 copies/μL (IQR, 13,045–40,000), whereas for the *ompB* assay and it was 19.25 copies/μL (IQR, 11.11-56.62) (Fig 3). Raw data of rickettsia target gene copy number are provided in S1 Data.

## Discussion

This study developed a qPCR assay targeting the *sca2* gene, which contains six repetitive sequences, for the detection of *R. typhi*. Unlike the single-copy *ompB* gene, this assay leverages multiple template copies per genome, enhancing analytical sensitivity for detecting low-level bacteremia commonly observed in murine typhus patients (approximately 200 copies/μL) [9]. A multiple-copy gene approach has been used for detection of various pathogens, including *Plasmodium falciparum* and *Plasmodium vivax* in asymptomatic infections [17]. Chao et al also developed a qPCR assay targeting multiple genes to detect fifteen different strains of *O. tsutsugamushi* isolates and in patient samples [10]. Although multiple gene copies are not found in the *R. typhi* genome, genes containing repetitive sequences have been identified [18]. In this study, we applied a multiple-target approach by designing primers and a probe to bind all six repetitive sequences within the *sca2* gene. This enabled the primers and probe to bind six times, compared to targeting a single-copy gene. Currently, qPCR assays for *R. typhi* detection in clinical samples typically target single sequence such as *ompB*. In our *in vitro* experiments using plasmid containing fragments of *sca2* and *ompB*, the *sca2*-based assay could detect DNA at 10-fold lower concentrations than the *ompB*-based assay (1copy/μL versus 10 copies/μL). In spiked samples, serial dilutions of *R. typhi* showed that the *sca2* assay had lower limit of detection (0.3 PFU) compared to *ompB* (3 PFU), indicating higher sensitivity. These findings were supported by accuracy testing using buffy coat samples with known *R. typhi* infection status. Another study used the parvulin-type PPIase (*prsA*) genes as the qPCR target and reported 10-fold greater sensitivity than *ompB [*7*]*. However, *prsA* testing was not evaluated in this study. Other *Rickettsia* genes, such as citrate synthase *(gltA)* and 17 kDa lipoprotein outer membrane antigens (17-kDa), were not evaluated in this study, as both assays were commonly used for genus-level screening; *ompB* and *sca2* were selected for species-specific detection [19]. Quantification of rickettsial target gene copy number using *sca2* and *ompB* assays revealed significantly higher bacterial copy numbers when using the *sca2*-based assay. This suggests that *sca2* is a valuable target for detecting *R. typhi* in patients with low bacterial loads and for monitoring bacteria clearance in antibiotic treatment studies. Interestingly, the rickettsia target gene copy number detected by *sca2* was approximately 800-fold higher than that determined by *ompB*, despite theoretical expectation of only a six-fold increase based on repeat copy number. Several factors may explain this discrepancy. First, the TaqMan probe can cleave a new probe during each PCR cycle, generating fluorescent signal more rapidly [20]. Second, secondary structures within the repeat sequences may make the amplicon more accessible, enhancing primer and probe binding [21]. Third, repeat sequences may amplify more efficiently than single-copy targets due to thermodynamic advantages [22]. Finally, in genes with tandem repeats, primers and probes can potentially rebind multiple times during extension, further increasing fluorescence [23].

Although *sca2* is more sensitive than *ompB* and can detect lower copy numbers in clinical samples with a low bacterial burden, the cost is not expected to be higher, as the assay differs only in primer sequences and uses the same probe labeling chemistry (5′ FAM and 3′ BHQ1). Cost would increase only if longer oligonucleotides are required.

The *sca2* qPCR assay is feasible for use in the tested population and can be applied alongside the *ompB* assay. Since murine typhus patients typically have low bacterial loads, *ompB*-based qPCR may fail to detect low levels of DNA. In such cases, *sca2* qPCR serves as a complementary target, improving diagnostic confidence.

Murine typhus patients are usually treated with doxycycline after rapid antibody-based testing, before qPCR confirmation. This does not differentially affect qPCR reaction [24], as antibiotic treatment reduces bacterial load and impacts detection of all targets similarly. Therefore, improved detection with *sca2* reflects enhanced analytical sensitivity at low bacterial loads rather than a gene-specific effect. In practice, qPCR positivity depends on the timing of sample collection relative to antibiotic initiation and the initial bacterial burden [25–27]. In this study, samples were collected at presentation or shortly after antibiotic therapy, reflecting routine diagnostic conditions.

This study had several limitations. Specificity testing was limited, as DNA from all *Rickettsia spp.* was not available. The number of clinical samples used for assay validation was small. The developed assay was optimized for detecting *R. typhi* in buffy coat samples, but not for other sample types or sources, including vectors and reservoirs. Additionally, only two assays were evaluated, and other genes with repeat sequences-such as those encoding ankyrin repeat-containing proteins, patatin-like phospoholipases were not included for comparison with *sca2.* Finally, rickettsial target gene copy number determined by *sca2* and *ompB* were calculated only from qPCR-positive samples, and thus do not represent all patients who tested positive by the gold standard method (IFA).

## Conclusion

We optimized a qPCR targeting *sca2* gene, which contains repetitive sequences for diagnosing *R. typhi* infection in humans. The assay is specific to *R. typhi* and does not cross-react with other *Rickettsia* species or a panel of other bacteria. *In vitro* studies using plasmid standards and bacteria spiked into human blood showed that the *sca2* assay had a limit of detection approximately 10-fold lower than that of *ompB*, the current commonly used qPCR target for *R. typhi* detection. This finding was supported by the sensitivity and specificity testing using patients’ stored buffy coat samples. Moreover, bacteria quantification using *sca2* yielded approximately 2.9 log higher values compared to *ompB* assay. Overall, the qPCR assay targeting *sca2* is a promising tool for diagnosis *R. typhi* in patients, who typically have low bacterial loads.

Future work will focus on optimizing the *sca2* assay for different sample types, including vectors and reservoirs, to support surveillance studies, as well as validating the assay with a larger set of clinical samples. We will also investigate other target genes containing repetitive sequences and compare their performance with *sca2* and *ompB*. Finally, we aim to use the selected target to develop a CRISPR-Cas–based lateral flow diagnostic assay and validate it with prospective patient samples.

## Supporting information

S1 Table List of stored buffy coat samples for sensitivity and specificity testing. Buffy coat samples with confirmed rickettsial infection by at least one method from culture or indirect immunofluorescence assay (IFA) or Rapid Diagnostic Test (RDT) or qPCR.
(DOCX)

S1 Fig Schematic of the *sca2* gene structure containing four regions containing repetitive sequences (green, yellow and blue) and the binding site of specific primer. (DOCX)

S2 Fig Gel electrophoresis analysis of DNA from sixteen laboratory strains of *R. typhi* and other bacteria using *sca2.* (DOCX)

S1 Data Dataset. **Raw data supporting the findings of this study.** This dataset contains raw qPCR Ct values for *sca2* and *ompB*, IFA titres (IgM and IgG) for patients confirmed by IFA, Ct values from spiked blood experiments, and bacterial loads measured by *sca2* and *ompB*. Each type of data is provided in separate sheet within Excel file.
(XLSX)
