# Supplementary material for: Targeting surface cell antigen 2 increases sensitivity of Rickettsia typhi detection
Source: PLoS Negl Trop Dis. 2026 Feb 19;20(2):e0014004. doi: 10.1371/journal.pntd.0014004 (PMC12919770; doi:10.1371/journal.pntd.0014004)
Supplement: S1 Table — Buffy coat samples with confirmed rickettsial infection by at least one method from culture or indirect immunofluorescence assay (IFA) or Rapid Diagnostic Test (RDT) or qPCR. (DOCX) [file pntd.0014004.s001.docx]

**S1 Table List of stored buffy coat samples for sensitivity and specificity testing**. Buffy coat samples with confirmed rickettsial infection by at least one method from culture or indirect immunofluorescence assay (IFA) or Rapid Diagnostic Test (RDT) or qPCR

| **No.** | **Sample Code** | **Pathogen** | **Scrub typhus or Murine typhus RDT** | **Culture** | **confirmed culture result by IFA** | ***Result for R. typhi or TG* detection** | **sca2-PCR** | ***ompB*-PCR** |
| --- | --- | --- | --- | --- | --- | --- | --- | --- |
| 1 | BC 1 | *O. tsutsugamushi* | Positive | Positive | Positive | Negative | Negative | Negative |
| 2 | BC 2 | *O. tsutsugamushi* | Positive | Positive | Positive | Negative | Negative | Negative |
| 3 | BC 3 | *O. tsutsugamushi* | Positive | Positive | Positive | Negative | Negative | Negative |
| 4 | BC 4 | *O. tsutsugamushi* | Positive | Positive | Positive | Negative | Negative | Negative |
| 5 | BC 5 | *O. tsutsugamushi* | Positive | Positive | Positive | Negative | Negative | Negative |
| 6 | BC 6 | *O. tsutsugamushi* | Positive | Positive | Positive | Negative | Negative | Negative |
| 7 | BC 7 | *O. tsutsugamushi* | Positive | Positive | Positive | Negative | Negative | Negative |
| 8 | BC 8 | *R. typhi* | Positive | Positive | Positive | Positive | **Positive** | **Positive** |
| 9 | BC 9 | *R. typhi* | Positive | Positive | Positive | Positive | **Positive** | **Positive** |
| 10 | BC 10 | *O. tsutsugamushi* | Positive | Positive | Positive | Negative | Negative | Negative |
| 11 | BC 11 | *O. tsutsugamushi* | Positive | Positive | Positive | Negative | Negative | Negative |
| 12 | BC 12 | *R. typhi* | Positive | Positive | Positive | Positive | **Positive** | Negative |
| 13 | BC 13 | *R. typhi* | Positive | Positive | Positive | Positive | **Positive** | **Positive** |
| 14 | BC 14 | *O. tsutsugamushi* | Positive | Positive | Positive | Negative | Negative | Negative |
| 15 | BC 15 | *O. tsutsugamushi* | Positive | Positive | Positive | Negative | Negative | Negative |
| 16 | BC 16 | *O. tsutsugamushi* | Positive | Positive | Positive | Negative | Negative | Negative |
| 17 | BC 17 | *O. tsutsugamushi* | Positive | Positive | Positive | Negative | Negative | Negative |
| 18 | BC 18 | *O. tsutsugamushi* | Positive | Positive | Positive | Negative | Negative | Negative |
| 19 | BC 19 | *O. tsutsugamushi* | Positive | Positive | Positive | Negative | Negative | Negative |
| 20 | BC 20 | *O. tsutsugamushi* | Positive | Positive | Positive | Negative | Negative | Negative |
| 21 | BC 21 | *O. tsutsugamushi* | Positive | Positive | Positive | Negative | Negative | Negative |
| 22 | BC 22 | *R. typhi* | Positive | Positive | Positive | Positive | **Positive** | **Positive** |
| 23 | BC 23 | *O. tsutsugamushi* | Positive | Positive | Positive | Negative | Negative | Negative |
| 24 | BC 24 | *O. tsutsugamushi* | Positive | Positive | Positive | Negative | Negative | Negative |
| **No.** | **Sample Code** | **Pathogen** | **Scrub typhus or Murine typhus RDT** | **Culture** | **confirmed culture result by IFA** | ***Result for R. typhi or TG* detection** | **sca2-PCR** | ***ompB*-PCR** |
| 25 | BC 25 | *O. tsutsugamushi* | Positive | Positive | Positive | Negative | Negative | Negative |
| 26 | BC 26 | *O. tsutsugamushi* | Positive | Positive | Positive | Negative | Negative | Negative |
| 27 | BC 27 | *O. tsutsugamushi* | Positive | Positive | Positive | Negative | Negative | Negative |
| 28 | BC 28 | *O. tsutsugamushi* | Positive | Positive | Positive | Negative | Negative | Negative |
| 29 | BC 29 | *R. typhi* | Positive | Positive | Positive | Positive | Negative | Negative |
| 30 | BC 30 | *O. tsutsugamushi* | Positive | Positive | Positive | Negative | Negative | Negative |
| 31 | BC 31 | *O. tsutsugamushi* | Positive | Positive | Positive | Negative | Negative | Negative |
| 32 | BC 32 | *O. tsutsugamushi* | Positive | Positive | Positive | Negative | Negative | Negative |
| 33 | BC 33 | *O. tsutsugamushi* | Positive | Positive | Positive | Negative | Negative | Negative |
| 34 | BC 34 | *O. tsutsugamushi* | Positive | Positive | Positive | Negative | Negative | Negative |
| 35 | BC 35 | *O. tsutsugamushi* | Positive | Positive | Positive | Negative | Negative | Negative |
| 36 | BC 36 | *O. tsutsugamushi* | Positive | Positive | Positive | Negative | Negative | Negative |
| 37 | BC 37 | *O. tsutsugamushi* | Positive | Positive | Positive | Negative | Negative | Negative |
| 38 | BC 38 | *O. tsutsugamushi* | Positive | Positive | Positive | Negative | Negative | Negative |
| 39 | BC 39 | *O. tsutsugamushi* | Positive | Positive | Positive | Negative | Negative | Negative |
| 40 | BC 40 | *O. tsutsugamushi* | Positive | Positive | Positive | Negative | Negative | Negative |
| 41 | BC 41 | *O. tsutsugamushi* | Positive | Positive | Positive | Negative | Negative | Negative |
| 42 | BC 42 | *O. tsutsugamushi* | Positive | Positive | Positive | Negative | Negative | Negative |
| 43 | BC 43 | *O. tsutsugamushi* | Positive | Positive | Positive | Negative | Negative | Negative |
| 44 | BC 44 | *R. typhi* | Positive | Positive | Positive | Positive | **Positive** | **Positive** |
| 45 | BC 45 | *O. tsutsugamushi* | Positive | Positive | Positive | Negative | Negative | Negative |
| 46 | BC 46 | *O. tsutsugamushi* | Positive | Positive | Positive | Negative | Negative | Negative |
| 47 | BC 47 | *O. tsutsugamushi* | Positive | Positive | Positive | Negative | Negative | Negative |
| 48 | BC 48 | *O. tsutsugamushi* | Positive | Positive | Positive | Negative | Negative | Negative |
| 49 | BC 49 | *O. tsutsugamushi* | Positive | Positive | Positive | Negative | Negative | Negative |
| 50 | BC 50 | *O. tsutsugamushi* | Positive | Positive | Positive | Negative | Negative | Negative |
| **No.** | **Sample Code** | **Pathogen** | **Scrub typhus or Murine typhus RDT** | **Culture** | **confirmed culture result by IFA** | ***Result for R. typhi or TG* detection** | **sca2-PCR** | ***ompB*-PCR** |
| 51 | BC 51 | *R. typhi* | Positive | Positive | Positive | Positive | **Positive** | **Positive** |
| 52 | BC 52 | *R. typhi* | Positive | Positive | Positive | Positive | **Positive** | **Positive** |
| 53 | BC 53 | *R. typhi* | Positive | Positive | Positive | Positive | Negative | Negative |
| 54 | BC 54 | *R. typhi* | Positive | Positive | Positive | Positive | **Positive** | **Positive** |
| 55 | BC 55 | *R. typhi* | Positive | Positive | Positive | Positive | Negative | Negative |
| 56 | BC 56 | *R. typhi* | Positive | Not done | Positive using paired serum | Positive | Negative | Negative |
| 57 | BC 57 | *R. typhi* | Positive | Not done | Positive using paired serum | Positive | Negative | Negative |
| 58 | BC 58 | *R. typhi* | Positive | Not done | Positive using paired serum | Positive | **Positive** | Negative |
| 59 | BC 59 | *R. typhi* | Positive | Not done | Positive using paired serum | Positive | Negative | Negative |
| 60 | BC 60 | *R. typhi* | Positive | Not done | Positive using paired serum | Positive | **Positive** | Negative |
| 61 | BC 61 | *R. typhi* | Positive | Not done | Positive using paired serum | Positive | Negative | Negative |
| 62 | BC 62 | *R. typhi* | Positive | Not done | Positive using paired serum | Positive | **Positive** | Negative |
| 63 | BC 63 | *R. typhi* | Positive | Not done | Positive using paired serum | Positive | Negative | Negative |
| 64 | BC 64 | *R. typhi* | Positive | Not done | Positive using paired serum | Positive | Negative | Negative |
| 65 | BC 65 | *R. typhi* | Positive | Not done | Positive using paired serum | Positive | **Positive** | Negative |
| 66 | BC 66 | *Non- O.tsutsugamushi and or R. typhi* | Negative | Not done | Not done | Negative | Negative | Negative |
| 67 | BC 67 | *Non- O.tsutsugamushi and or R. typhi* | Negative | Not done | Not done | Negative | Negative | Negative |
| 68 | BC 68 | *Non- O.tsutsugamushi and or R. typhi* | Negative | Not done | Not done | Negative | Negative | Negative |
| 69 | BC 69 | *Non- O.tsutsugamushi and or R. typhi* | Negative | Not done | Not done | Negative | Negative | Negative |
| **No.** | **Sample Code** | **Pathogen** | **Scrub typhus or Murine typhus RDT** | **Culture** | **confirmed culture result by IFA** | ***Result for R. typhi or TG* detection** | **sca2-PCR** | ***ompB*-PCR** |
| 70 | BC 70 | *Non- O.tsutsugamushi and or R. typhi* | Negative | Not done | Not done | Negative | Negative | Negative |
| 71 | BC 71 | *Non- O.tsutsugamushi and or R. typhi* | Negative | Not done | Not done | Negative | Negative | Negative |
| 72 | BC 72 | *Non- O.tsutsugamushi and or R. typhi* | Negative | Not done | Not done | Negative | Negative | Negative |
| 73 | BC 73 | *Non- O.tsutsugamushi and or R. typhi* | Negative | Not done | Not done | Negative | Negative | Negative |
| 74 | BC 74 | *Non- O.tsutsugamushi and or R. typhi* | Negative | Not done | Not done | Negative | Negative | Negative |
| 75 | BC 75 | *Non- O.tsutsugamushi and or R. typhi* | Negative | Not done | Not done | Negative | Negative | Negative |
| 76 | BC 76 | *Non- O.tsutsugamushi and or R. typhi* | Negative | Not done | Not done | Negative | Negative | Negative |
| 77 | BC 77 | *Non- O.tsutsugamushi and or R. typhi* | Negative | Not done | Not done | Negative | Negative | Negative |
| 78 | BC 78 | *Non- O.tsutsugamushi and or R. typhi* | Negative | Not done | Not done | Negative | Negative | Negative |
| 79 | BC 79 | *Non- O.tsutsugamushi and or R. typhi* | Negative | Not done | Not done | Negative | Negative | Negative |
| 80 | BC 80 | *Non- O.tsutsugamushi and or R. typhi* | Negative | Not done | Not done | Negative | Negative | Negative |
| 81 | BC 81 | *Non- O.tsutsugamushi and or R. typhi* | Negative | Not done | Not done | Negative | Negative | Negative |
| 82 | BC 82 | *Non- O.tsutsugamushi and or R. typhi* | Negative | Not done | Not done | Negative | Negative | Negative |
