# Supplementary material for: Targeting surface cell antigen 2 increases sensitivity of Rickettsia typhi detection
Source: PLoS Negl Trop Dis. 2026 Feb 19;20(2):e0014004. doi: 10.1371/journal.pntd.0014004 (PMC12919770; doi:10.1371/journal.pntd.0014004)
Supplement: S1 Fig — (DOCX) [file pntd.0014004.s002.docx]

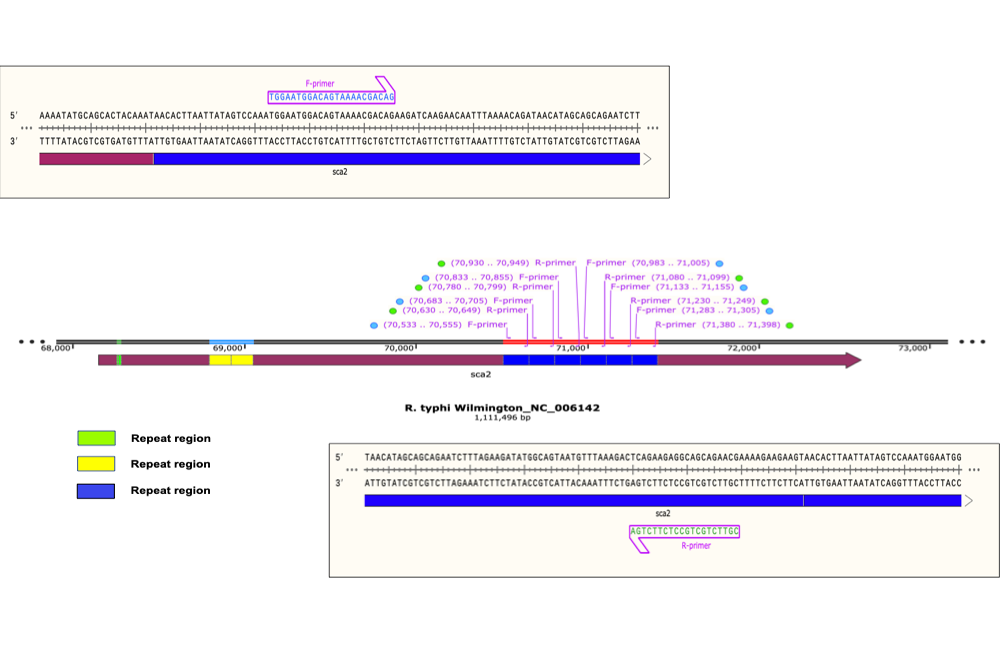


**S1 Fig Schematic of the sca2 gene structure containing four regions containing repetitive sequences (green, yellow and blue) and the binding site of specific primer**. The yellow regions contain two repetitive sequences, where the shorter sequence is a subset or overlaps with the longer sequence. The blue region contains the highest number of repetitive sequences, with six repeats. The forward and reverse primers were designed to target a conserved sequence encompassing six binding positions. The schematic of sca2 was illustrated by SnapGene (version 8.0.3)
