# Supplementary material for: Targeting surface cell antigen 2 increases sensitivity of Rickettsia typhi detection
Source: PLoS Negl Trop Dis. 2026 Feb 19;20(2):e0014004. doi: 10.1371/journal.pntd.0014004 (PMC12919770; doi:10.1371/journal.pntd.0014004)
Supplement: S2 Fig — (DOCX) [file pntd.0014004.s003.docx]

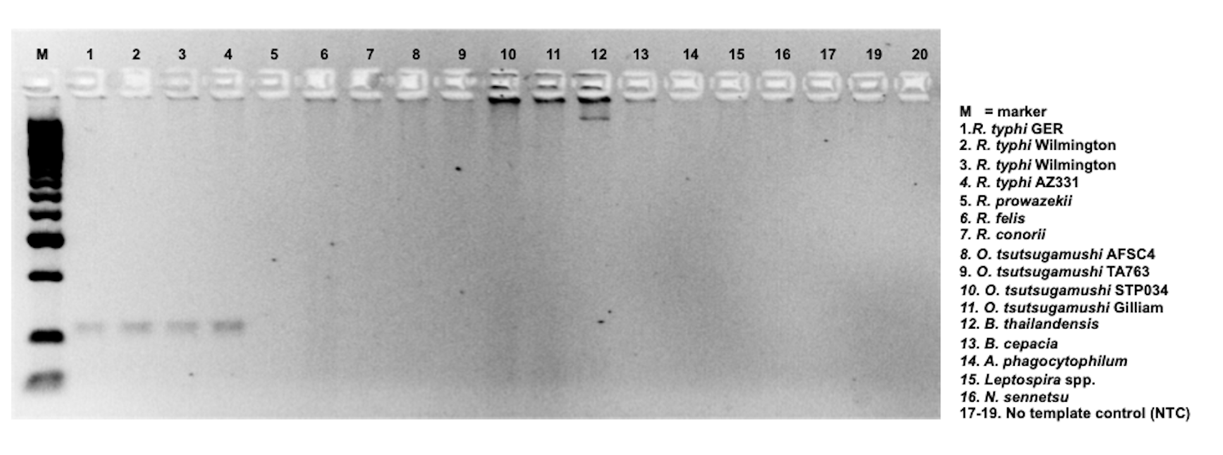


**S2 Fig Gel electrophoresis analysis of DNA from sixteen laboratory strains of R. typhi and other bacteria using sca2.**
